# Outbreak-associated *Salmonella* Baildon found in wastewater demonstrates how sewage monitoring can supplement traditional disease surveillance

**DOI:** 10.1128/jcm.00825-24

**Published:** 2024-09-19

**Authors:** Nkuchia M. M'ikanatha, Zoe S. Goldblum, Nicholas Cesari, Erin M. Nawrocki, Yezhi Fu, Jasna Kovac, Edward G. Dudley

**Affiliations:** 1 Division of Infectious Disease Epidemiology, Pennsylvania Department of Health, Harrisburg, Pennsylvania, USA; 2 Center for Clinical Epidemiology and Biostatistics, Perelman School of Medicine, University of Pennsylvania, Philadelphia, Pennsylvania, USA; 3 Department of Food Science, The Pennsylvania State University, University Park, Pennsylvania, USA; 4 The E. coli Reference Center, The Pennsylvania State University, University Park, Pennsylvania, USA; Maine Medical Center Department of Medicine, Portland, Maine, USA

**Keywords:** *Salmonella*, communicable disease, food safety, foodborne infections, disease outbreaks, foodborne pathogens, domestic wastewater surveillance, epidemiological monitoring

## Abstract

Non-typhoidal *Salmonella* is a common cause of gastroenteritis worldwide, but current non-typhoidal *Salmonella* surveillance is suboptimal. Here, we evaluated the utility of wastewater monitoring to enhance traditional surveillance for this foodborne pathogen. In June 2022, we tested raw sewage collected twice a week from two treatment plants in central Pennsylvania for non-typhoidal *Salmonella* and characterized isolates using whole-genome sequencing. We recovered 43 *Salmonella* isolates from wastewater samples, differentiated by genomic analysis into seven serovars: 16 Panama (37.2%), 9 Senftenberg (20.9%), 8 Baildon (18.6%), and 3 or fewer of four other serovars. We assessed genetic relatedness and epidemiologic links between these wastewater isolates with those from patients with salmonellosis. All *S*. Baildon isolates from wastewater were genetically similar to those associated with a known contemporaneous salmonellosis outbreak. *S*. Baildon from wastewater and 42 outbreak-related isolates in the national outbreak detection database had the same core genome multilocus sequence typing, and outbreak code differed by zero or one single polynucleotide polymorphism. One of the 42 outbreak-related isolates was obtained from a patient residing in the wastewater sample collection catchment area, which serves approximately 17000 people. *S*. Baildon is a rare serovar (reported in <1% cases nationally, over five years). Our study underscores the value of monitoring sewage from a defined population to supplement traditional surveillance methods for the evidence of *Salmonella* infections and to determine the extent of outbreaks.

**IMPORTANCE:**

During the COVID-19 pandemic, monitoring for SARS-CoV-2 in wastewater was highly effective in identifying the variants of concern earlier than clinical surveillance methods. Here, we show that monitoring domestic sewage can also augment traditional reporting of foodborne illnesses to public health authorities. Our study detected multiple *Salmonella enterica* serovars in samples from two wastewater treatment plants in central Pennsylvania. Using whole-genome sequencing, we demonstrated that the isolates of variant *S*. Baildon clustered with those from a foodborne salmonellosis outbreak that occurred in a similar time frame. Cases were primarily from Pennsylvania, and one individual lived within the wastewater treatment catchment area. This study highlights the effectiveness of domestic sewage testing as a proactive public health strategy to track and respond to infectious disease outbreaks.

## INTRODUCTION

Non-typhoidal *Salmonella* is a common cause of gastroenteritis globally, but current surveillance systems are inadequate. In the United States, reporting relies heavily on healthcare professionals and laboratories, yet underreporting, incomplete data, and limited resources hinder the effective detection of outbreaks (1–4). An estimated 1.03 million cases occur annually in the US, with only 52,575 reported in 2023 through the National Notifiable Diseases Surveillance System (https://wonder.cdc.gov/nndss/static/2023/52/2023-52-table1122.html). The burden of salmonellosis is significant, resulting in 74,000 physician visits, 19,000 hospitalizations, 378 deaths, and $4.14 billion in direct and indirect costs annually in the US (https://www.ers.usda.gov/data-products/cost-estimates-of-foodborne-illnesses/). The substantial underreporting through traditional surveillance systems, coupled with the high burden of illness and economic impact, underscores the need for improved methods to monitor non-typhoidal *Salmonella* infections.

For diseases such as salmonellosis and COVID-19, unrecognized cases can amplify transmission (5, 6). Evidence of infection can be detected in stool samples from approximately 90% of individuals infected with *Salmonella* and 40% of those with SARS-CoV-2 (7, 8). Identification and quantification of SARS-CoV-2 RNA in untreated influents from domestic wastewater treatment plants augmented traditional disease reporting during the pandemic and is now a major component of COVID-19 surveillance in the United States (9). Wastewater-based surveillance detects SARS-CoV-2 RNA before the peak of clinical cases and has been shown to identify variants of concern before presentation in patients (10, 11). Emergency appropriations in 2021 expanded wastewater-based surveillance efforts for COVID-19 through the US Food and Drug Administration (FDA) GenomeTrakr program, which has been focused on foodborne pathogens (12).

We participated in the GenomeTrakr COVID-19 wastewater testing and reasoned that similar monitoring could be useful for non-typhoidal *Salmonella* surveillance. The Pennsylvania Department of Health Bureau of Laboratories (BOL) performs whole-genome sequencing of non-typhoidal *Salmonella* found in clinical samples and uploads data to the Centers for Disease Control and Prevention (CDC) PulseNet program to facilitate outbreak investigations (13). Diagnostic laboratories statewide send presumptive *Salmonella* isolates to BOL in compliance with communicable disease regulations, which also mandate the reporting of detailed patient information, including demographic characteristics, via the secure Pennsylvania National Electronic Disease Reporting system (PA-NEDSS) (14). Investigations of individual salmonellosis cases and outbreaks are documented in PA-NEDSS (15). Additionally, Pennsylvania sends some isolates from patients and all non-typhoidal *Salmonella* isolates found in retail food sources to the National Antimicrobial Resistance Monitoring System (NARMS) laboratories at the CDC and the FDA, respectively (16).

By leveraging the SARS-CoV-2 variants project (12) and the existing state and national public health systems for salmonellosis (13–16), we conducted a study to assess whether wastewater-based surveillance could enhance surveillance for non-typhoidal *Salmonella*. We hypothesized that *Salmonella* isolates linked to recent outbreaks would be detected in domestic sewage.

## MATERIALS AND METHODS

### Catchment area population and characteristics

Wastewater samples were collected from two treatment facilities located in a predominantly rural region of central Pennsylvania. A wastewater treatment plant manager in each facility completed a brief survey that included information on the number of customers and presence of institutional residential facilities such as prisons within the catchment area. We used the household equivalent (customers served) combined with United States Census Bureau data to estimate the total population of each catchment area (https://www.census.gov/quickfacts/fact/table/PA/HSG010222). Twice weekly during 13–29 June 2022, a plant operator at each facility collected and provided approximately 1 L of a flow-proportional 24 h untreated sewage composite sample. The composite sample was obtained using a sampler programed to take 500 mL volumes for each 50,000 gallons of wastewater entering the plant, upstream of the primary clarifier following the United States Environmental Protection Agency protocols (https://www.epa.gov/quality/procedures-collecting-wastewater-samples). Samples were stored at 4°C in the dark for less than 48 h before being transported on ice to the laboratory for processing.

### Detection of *Salmonella* in raw wastewater

Briefly, we transferred 120 mL of wastewater into a bottle, centrifuged at 5,000× *g* for 20 min at 4°C, filtered the supernatant through a 0.45 µm membrane, and added the filter to 40 mL of buffered peptone water supplemented with 20 mg/L novobiocin. Broths were incubated at 35°C for 24 h. Selective enrichment and selective plating were performed according to the FDA Bacteriological Analytical Manual (BAM) (17).

Concisely, 1 mL of peptone enrichment was inoculated into 9 mL of tetrathionate broth and 0.1 mL into 9.9 mL of RVS broth and incubated at 42°C for 24 h. Aliquots of 10 µL of each enriched culture were then plated on Xylose Lysine Deoxycholate (XLD) and Hektoen Enteric (HE) agar. Eight typical colonies (blue-green to blue colonies with or without black centers on HE, pink colonies with or without black centers on XLD) were selected, and streaked on the alternate medium, and grown again overnight. All isolates grew on both media. Isolates with colony morphologies consistent with *Salmonella* on both media were screened for *invA* by PCR (18). Forty-three were confirmed to be *Salmonella*, and thus whole-genome sequenced (Table S1).

### Whole-genome sequencing and cluster detection

We extracted DNA from *Salmonella* isolates with the Qiagen DNeasy blood and tissue kit, and libraries were prepared using the Nextera XT DNA library kit. Libraries were sequenced using an Illumina MiSeq using the manufacturer’s reagent kits (MiSeq V3 kit) with 500 (2 × 250) cycles. We uploaded short-read DNA sequences to the NCBI Pathogen Detection Isolate Browser (NCBI-PD) to automatically analyze and display the relationships among pathogens. The relationship is based on single-nucleotide polymorphism (SNP) variations in DNA between individual organisms (https://www.ncbi.nlm.nih.gov/pathogens/pathogens_help/#isolates-browser). The SeqSero2 application within the NCBI-PD pipeline was used to identify molecular serotypes (19). Additionally, we used the NCBI-PD pipeline AMRFinderPlus to identify antimicrobial resistance gene sequences and mutations that matched with those in the Reference Gene Catalog (20).

### Verification of clusters and epidemiological investigations

We used the CDC Systems Enteric Disease Response, Investigation, and Coordination (SEDRIC) platform to verify clustering of isolates from clinical and wastewater sources (21). SEDRIC analyses are based on core genome multilocus sequence typing, which is a gene-by-gene comparison approach used for outbreak cluster detection. We considered isolates from clinical and wastewater sources related if they differed by ≤10 alleles (22). SEDRIC enabled us to pinpoint each isolate by geographic location, temporality, allele code, and associated multi-state outbreak code, if any. For related isolates from Pennsylvania, we reviewed original records in PA-NEDSS. We also examined summaries of outbreaks including demographic and epidemiological characteristics. To assess the prevalence of *S*. Baildon, we analyzed reported salmonellosis cases from the catchment area between 2019 and 2022 as well as data available in state and national surveillance systems. Additionally, we compared *S*. Baildon isolate sequences with those of all nontyphoidal *Salmonella* cases reported in the catchment area, Pennsylvania, and the entire United States.

### Evolutionary analysis by the maximum likelihood method

We analyzed the genetic relatedness of isolates from clinical and wastewater sources using the FDA Center for Food Safety and Applied Nutrition (CFSAN) SNP Pipeline on the GalaxyTrakr website as previously described (23). The generated output snp_profile.fasta file was used to build a maximum likelihood tree using the MEGA11 (Molecular Evolutionary Genetic Analysis) application (24, 25).

## RESULTS

### Catchment area characteristics

There were 5,600 and 1,500 equivalent households served by wastewater treatment plants A and B, respectively. The average persons per household in the state in 2022 was 2.42 (https://www.census.gov/quickfacts/fact/table/PA/HSG010222). Thus, the total population in the catchment area of the two Pennsylvania wastewater treatment plants, from which samples were collected, was estimated at 17,182. The estimated population in the two facilities with high-risk for salmonellosis was 679 [139 and 540 at the Clearfield County Jail and the Moshannon Valley Processing Center, respectively (https://pennsylvaniaprisonroster.org/pennsylvania/county-jail/clearfield-county-prison/, https://www.dhs.gov/sites/default/files/2022-10/OIDO%20Final%20Inspection%20Report%20-%20Moshannon%20Valley%20Processing%20Center_2.pdf)]. The two wastewater treatment plants receive domestic sewage only, without any storm water runoff or environmental runoff such as that from farm operations or poultry processing plants.

### *Salmonella* serovars isolated from wastewater

**FIG 1:**
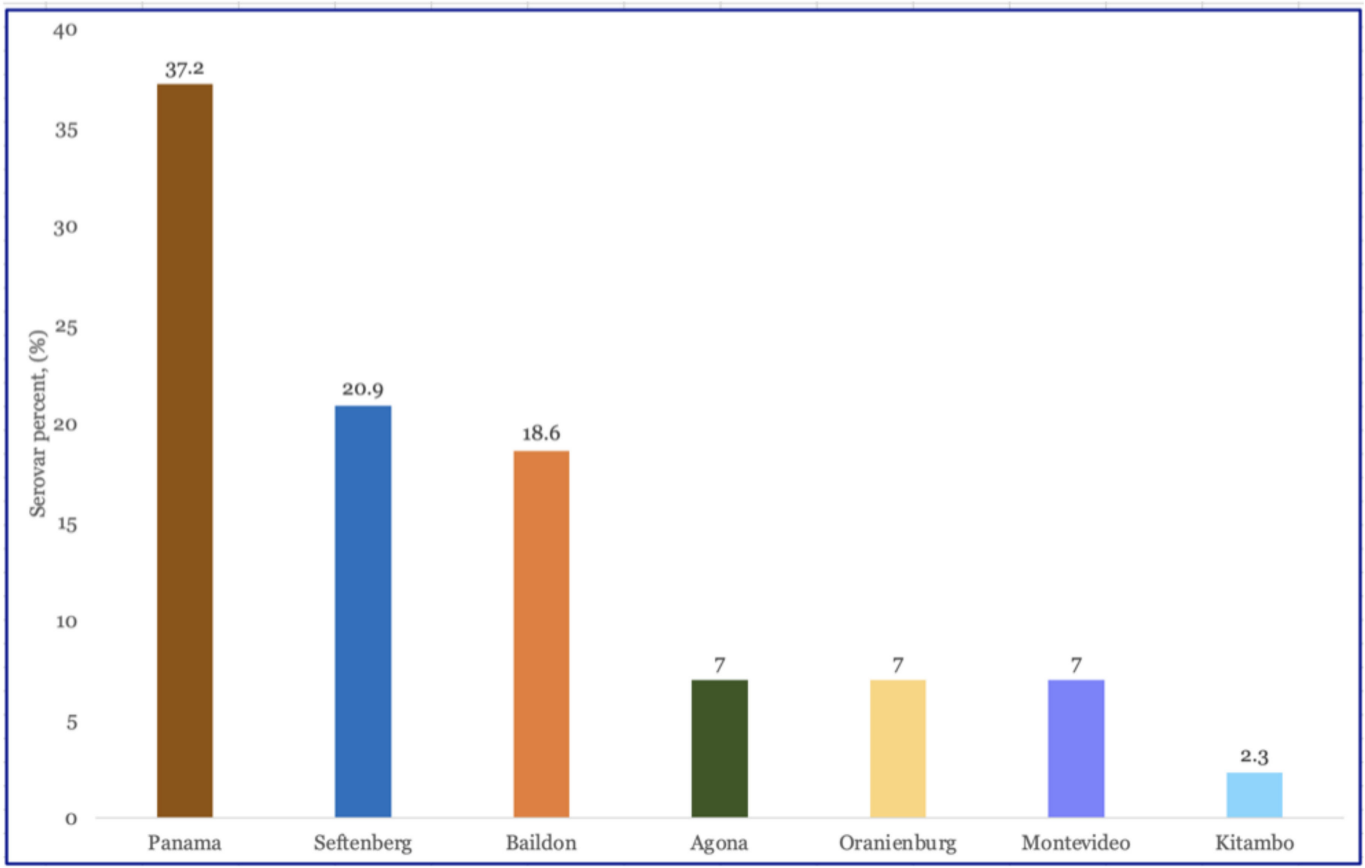
Distribution of non-typhoidal *Salmonella* serotypes identified among the 43 isolates collected during June 2022. The catchment area served by two wastewater treatment plants in central Pennsylvania served a total population of approximately 17,182.

*Salmonella* was found in 9 of the 12 wastewater samples (75%) that were collected from the Pennsylvania plants in June 2022; 43 *Salmonella* isolates were recovered. As previously reported (26), based on whole-genome assemblies, these isolates were from seven serovars: 16 were Panama (37.2%), 9 were Senftenberg (20.9%), and 8 were Baildon (18.6%). Four other serovars had three or fewer isolates (Fig. 1). There were no clinically relevant antimicrobial resistance genes or resistance-conferring SNPs identified in any of the isolates.

**FIG 2:**
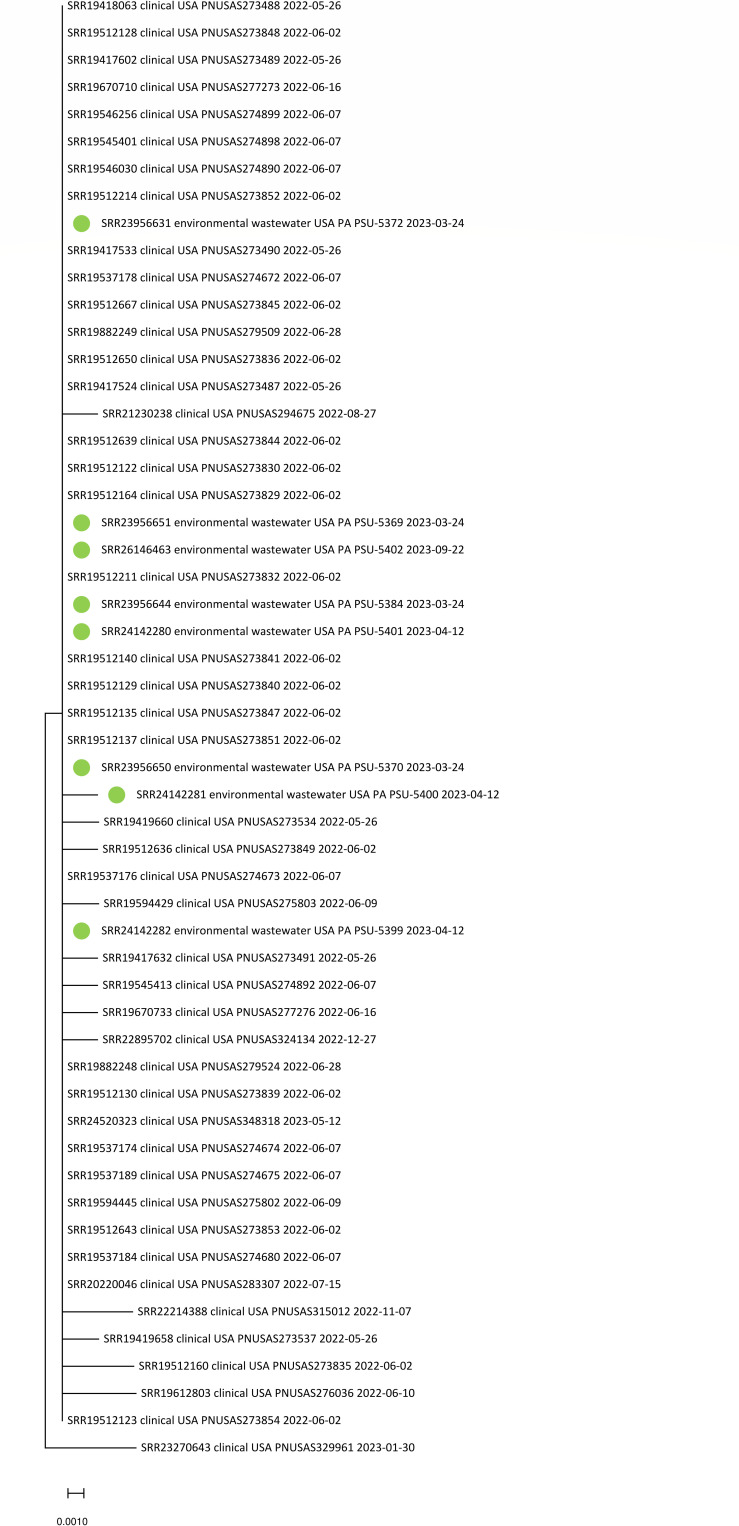
Genetic relatedness of *S*. Baildon isolates (*n* = 8) from sewage sources (marked green) and clinical sources (*n* = 46). Isolates from sewage samples were separated by 0 or 1 SNP from those linked to salmonellosis. The tree is drawn to scale, with branch lengths measured in the number of substitutions per site. Evolutionary analyses were conducted in MEGA11 (27).

**FIG 3:**
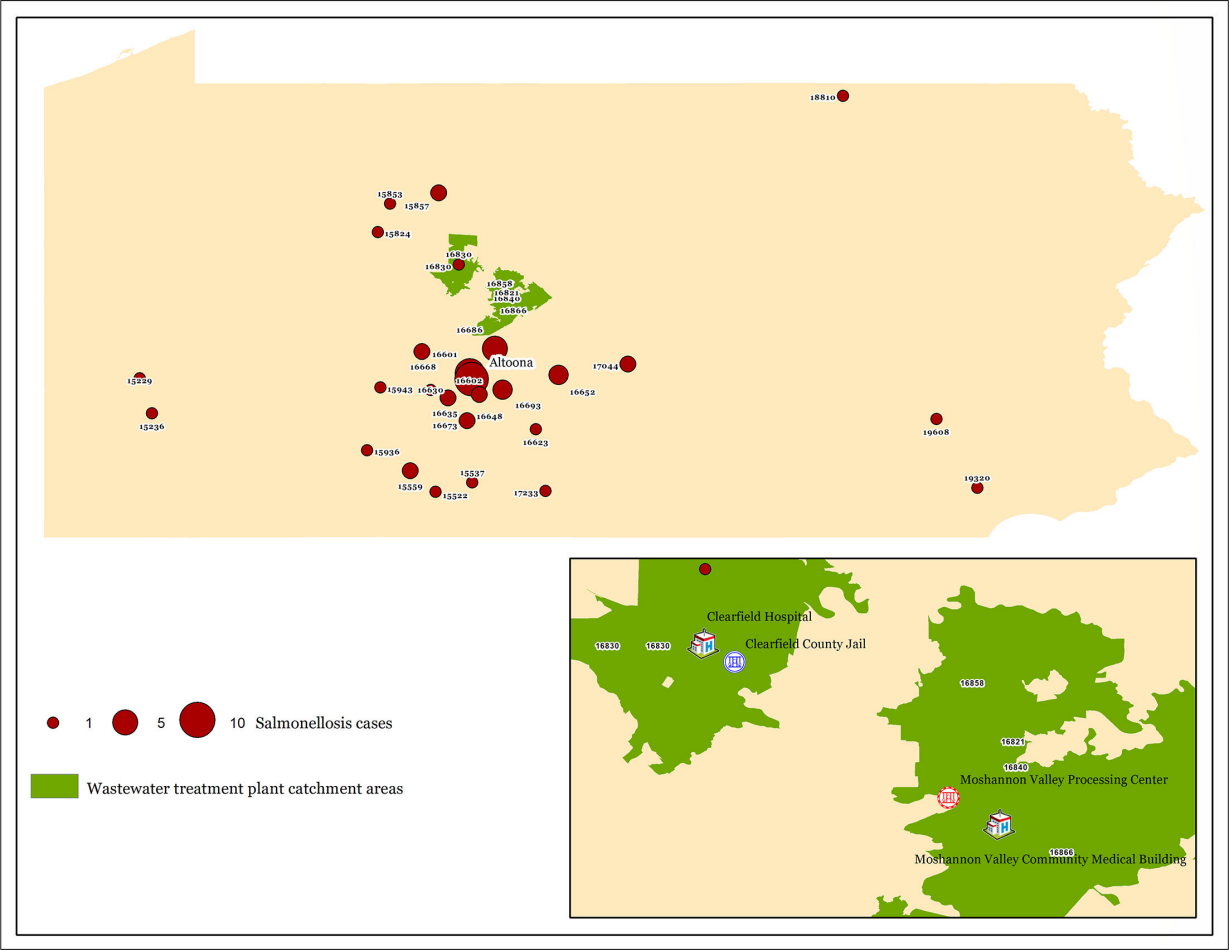
Locations of domestic sewage treatment plant catchment areas (green) and locations of zip codes where clinical cases were reported with numbers of cases indicated by sizes of the red dots. Also indicated are the location of the Clearfield County Jail, and Moshannon Valley Processing Center, which in addition to community households are included in the catchment area. Hospitals are included to show that the area has health care facilities. Wastewater from hospitals is not allowed in domestic wastewater treatment plants by Pennsylvania and federal regulations.

The eight *S*. Baildon isolates were recovered on two separate days from one wastewater treatment facility and on three separate days from the other. All 8 (100%) *S*. Baildon isolates from domestic sewage samples were separated by 0 or 1 SNPs from 46 clinical isolates identified in SEDRIC (Fig. 2), and all were assigned the same allele code in the national outbreak database. Forty-four (95.7%) of the clinical isolates originated from Pennsylvania, and two (SRR19419658 and SRR19512122) were from nearby states (West Virginia and Virginia) (Table S2). One of the Pennsylvania *S*. Baildon isolates was from a patient residing within the catchment area (Fig. 3). Although the clinical isolates are in the Centers for Disease Control database (SEDRIC), the agency does not typically declare outbreaks primarily in one jurisdiction.

### Epidemiological investigation of genetically related *S.* Baildon isolates from wastewater and clinical sources

Forty-two (91.3%) of the 46 clinical *Salmonella* isolates were linked to a salmonellosis outbreak in Pennsylvania from 3 to 5, May 2022. This linkage was established based on the outbreak case definition. Epidemiological evidence strongly suggests the outbreak was associated with the consumption of contaminated tomato and iceberg lettuce (odds ratio 7.25, 95% confidence interval 1.98–26.54; *P* = 0.003). The food was consumed at a chain restaurant in Altoona, Pennsylvania, within an hour’s driving distance from the wastewater catchment areas (Fig. 3).

Our review of state-based surveillance found that during the 4-year period 2019–2023, only 11 salmonellosis cases were reported from the wastewater catchment area. During the same 4-year period, *S*. Baildon was associated with only 53 (0.7%) of the 8,143 cases of salmonellosis in Pennsylvania and only 650 (0.2%) of 330,172 cases in the United States (Table 1).

**TABLE 1:** Prevalence of *Salmonella* Baildon in Pennsylvania and United States—2019–2023^*a*^

Year	Cases reported by jurisdiction					
	Pennsylvania		Catchment area		United States	
	NTS^*b*^	Baildon	NTS	Baildon	NTS	Baildon
2019	1,574	1	1	0	73,864	126
2020	1,580	2	2	0	58,930	137
2021	1,457	0	3	0	64,357	119
2022	1,768	47		1	71,528	143
2023	1,764	3	5	0	61,493	125
Total	8,143	53		1	330,172	650
Percent		0.65				0.97

^*a*^ Source: SEDRIC (26) and PA-NEDSS (17).

^*b*^ NTS, non-typhoidal *Salmonella*.

## DISCUSSION

This study demonstrated that monitoring wastewater can complement traditional, patient-encounter-based monitoring systems for non-typhoidal *Salmonella*. Non-typhoidal *Salmonella* strains were isolated from raw domestic sewage collected from two facilities serving approximately 17,000 residents in central Pennsylvania. Whole-genome sequencing of the 43 non-typhoidal *Salmonella* strains identified in raw sewage samples revealed seven serotypes. Among isolates from domestic wastewater, two serotypes (*S*. Senftenberg and *S*. Baildon) were genetically linked to recent clinical cases. The *S*. Senftenberg isolates were similar to those from a 2022 multistate outbreak linked to contaminated peanut butter as previously reported (26). The current study focuses on the *S*. Baildon isolates, which clustered with strains associated with a foodborne outbreak predominantly reported in Pennsylvania. Infections associated with both outbreaks were reported to public health authorities either concurrently with or shortly after *Salmonella* was detected in wastewater samples from the corresponding catchment area.

The eight S. Baildon isolates were indistinguishable from *Salmonella* detected in patients from or close to the wastewater catchment area who likely were infected by consumption of contaminated tomato and iceberg salad at a restaurant located approximately 50 miles from our catchment area. *S*. Baildon infections are primarily acquired through the consumption of contaminated salads (28). Notably, since its inception in 1997, the FDA NARMS retail food program (monitors enteric bacteria in various types of meat and sea food samples) has not detected *S*. Baildon in over 200,000 samples tested (data not shown). Moreover, although antimicrobial resistance genes are frequently found in *Salmonella* isolated from food of animal origins, particularly poultry (https://www.fda.gov/animal-veterinary/national-antimicrobial-resistance-monitoring-system/narms-now-integrated-data), these genes were absent in the *S*. Baildon isolated from wastewater in this study.

That isolates associated with clinical salmonellosis cases can be detected in wastewater was previously demonstrated. For example, a study in Hawaii identified *S*. Derby strains in wastewater that were linked to asymptomatic cases not detected by traditional surveillance (29). In Texas, wastewater testing successfully identified the causative agent, *S*. Heidelberg, during and after a 2003 salmonellosis outbreak (30). Similarly, Sahlström et al. found genetically matched *Salmonella* serotypes in wastewater and clinical salmonellosis cases in Sweden (27). Additionally, Berge *et al.* observed differences in *Salmonella* serovars from wastewater treatment plants and patient samples in California, suggesting the presence of unreported infections (31). These studies, along with our own, highlight the potential of wastewater surveillance as a valuable tool to complement existing public health monitoring for *Salmonella*.

Although feces from pets and from rodents infected with *Salmonella* may enter the domestic sewage stream, the *S*. Baildon isolates in this study most likely originated from humans based on several lines of evidence. First, the genetic linkage of these isolates to serovars associated with outbreaks strongly suggests a human, rather than animal, reservoir. Second, the low reported prevalence of *S*. Baildon (associated with <1% of salmonellosis cases in the United States over the past 5 years) supports this hypothesis. Third, epidemiological investigations have consistently linked *S*. Baildon outbreaks to the consumption of contaminated food, with possible human-to-human transmissions. Notably, two multi-state outbreaks associated with *S*. Baildon, occurring a decade apart in 1998 and 2008, were both traced back to contaminated food in restaurants (27 and https://archive.cdc.gov/#/details?url=https://www.cdc.gov/salmonella/2010/restaurant-chain-a-8-4-10.html. Therefore, although *Salmonella* does infect animals, the evidence strongly suggests that the *S*. Baildon isolates found in sewage samples in this study originated from humans. Studies on the prevalence of zoonotic serovars in household pets and rodents, and their presence in domestic wastewater, could enhance sewage monitoring reliability.

The finding that the most genetically similar *S*. Baildon clinical isolates were from cases from an outbreak that occurred near the study’s wastewater catchment area in the same time frame as our sample collection is noteworthy. Outbreak investigations often underestimate the true number of cases, and the precise geographic spread remains unclear (28, 32). Severe *Salmonella* infections, including those caused by *S*. Baildon, are more likely to be reported to public health authorities than mild or asymptomatic cases (1–4, 28). The CDC estimates that traditional surveillance methods only identify 1 in 29 *Salmonella* infections (32). Furthermore, foodborne illnesses are common, particularly in institutional settings such as prisons and nursing homes, but they are often under-reported (33, 34). This highlights the significant shortcomings of reliance on patient encounters with healthcare providers for salmonellosis surveillance and underscores the need for improved surveillance methods.

Our study was limited by the collection of samples from only two wastewater plants, both convenient to our laboratory. However, the catchment area population is comparable to those in the typical rural municipalities in Pennsylvania, which are home to about 26% of the state’s 13.0 million residents (https://www.rural.pa.gov/data/rural-quick-facts). The study’s short duration of less than a month is also a limitation, as *Salmonella* isolation rates likely fluctuate over time. Inherent uncertainties also exist in surveillance data because most infectious diseases including those caused by *Salmonella* are under-reported. Additionally, although the testing of domestic sewage without stormwater increased the likelihood that detected *Salmonella* originated from humans, non-human sources cannot be definitively excluded.

In summary, our findings, combined with previous studies from diverse settings (27–30), highlight the potential of domestic sewage monitoring to address weaknesses in traditional surveillance systems (3, 4, 32) This approach could enhance current resource-intensive patient-based systems, which often lack sensitivity and timeliness (35–37). Targeted monitoring for enteric pathogens in sewage could complement existing methods, with populations selected based on surveillance findings. For example, testing for *Salmonella* in sewage originating from institutions where the risk of foodborne is high, such as prisons and nursing homes (33, 34), could be especially valuable. Our study also demonstrates the power of pathogen genomics, utilized through partnerships between public health jurisdictions and academia, to enhance *Salmonella* surveillance.

## Data Availability

Sequence short reads for all wastewater isolates were uploaded to the NCBI under BioProject PRJNA357723. Accession numbers are provided in Tables S1 and S2 for isolates from sewage and clinical sources, respectively.

## SUPPLEMENTAL MATERIAL

Table S1 jcm.00825-24-s0001.pdf Outbreak-associated *Salmonella* Baildon found in wastewater.

Table S2 jcm.00825-24-s0002.pdf Outbreak-associated *Salmonella* Baildon detected in wastewater and clinical isolates.

ASM does not own the copyrights to Supplemental Material that may be linked to, or accessed through, an article. The authors have granted ASM a non-exclusive, world-wide license to publish the Supplemental Material files. Please contact the corresponding author directly for reuse.
