# Supplementary material for: Outbreak-associated Salmonella Baildon found in wastewater demonstrates how sewage monitoring can supplement traditional disease surveillance
Source: J Clin Microbiol. 2024 Sep 19;62(10):e00825-24. doi: 10.1128/jcm.00825-24 (PMC11481576; doi:10.1128/jcm.00825-24)
Supplement: Table S1 — Outbreak-associated Salmonella Baildon found in wastewater. [file jcm.00825-24-s0001.pdf]

Supplemental Table 1: Outbreak-associated *Salmonella* Baildon found in wastewater

Strain Names	PSU Name	bioproject_accession	SAMN	SRA	Serotype
C.S.6.16.22.1	PSU-5402	PRJNA357723	SAMN37513825	SRR26146463	Baildon
C.S.6.16.22.2	PSU-5396	PRJNA357723	SAMN34154794	SRR24142285	Senftenberg
C.S.6.16.22.3	PSU-5369	PRJNA357723	SAMN33902324	SRS17129852	Baildon
C.S.6.16.22.6	PSU-5397	PRJNA357723	SAMN34154795	SRR24142284	Senftenberg
C.S.6.16.22.5	PSU-5398	PRJNA357723	SAMN34154796	SRR24142283	Senftenberg
C.S.6.16.22.4	PSU-5370	PRJNA357723	SAMN33902325	SRS17129853	Baildon
C.S.6.15.22.1	PSU-5371	PRJNA357723	SAMN33902326	SRS17129864	Kintambo
C.S.6.15.22.2	PSU-5372	PRJNA357723	SAMN33902327	SRS17129872	Baildon
P.S.6.15.22.1	PSU-5373	PRJNA357723	SAMN33902328	SRS17129873	Panama
P.S.6.15.22.2	PSU-5374	PRJNA357723	SAMN33902329	SRS17129874	Panama
P.S.6.15.22.3	PSU-5375	PRJNA357723	SAMN33902330	SRS17129875	Senftenberg
P.S.6.15.22.4	PSU-5376	PRJNA357723	SAMN33902331	SRS17129876	Senftenberg
P.S.6.15.22.5	PSU-5377	PRJNA357723	SAMN33902332	SRS17129877	Senftenberg
P.S.6.15.22.6	PSU-5399	PRJNA357723	SAMN34154797	SRR24142282	Baildon
P.S.6.13.22.1	PSU-5378	PRJNA357723	SAMN33902333	SRS17129878	Agona
P.S.6.13.22.2	PSU-5379	PRJNA357723	SAMN33902334	SRS17129854	Agona
P.S.6.13.22.3	PSU-5400	PRJNA357723	SAMN34154798	SRR24142281	Baildon
P.S.6.13.22.4	PSU-5401	PRJNA357723	SAMN34154799	SRR24142280	Baildon
P.S.6.13.22.5	PSU-5403	PRJNA357723	SAMN37513826	SRR26146462	Senftenberg
P.S.6.13.22.6	PSU-5404	PRJNA357723	SAMN37513827	SRR26146461	Agona
P.S.6.13.22.7	PSU-5405	PRJNA357723	SAMN37513828	SRR26146460	Senftenberg
P.S.6.20.22.1	PSU-5380	PRJNA357723	SAMN33902335	SRS17129855	Panama
P.S.6.20.22.2	PSU-5406	PRJNA357723	SAMN37513829	SRR26146459	Panama
P.S.6.20.22.3	PSU-5407	PRJNA357723	SAMN37513830	SRR26146458	Panama
P.S.6.20.22.4	PSU-5381	PRJNA357723	SAMN33902336	SRS17129856	Panama
P.S.6.20.22.5	PSU-5382	PRJNA357723	SAMN33902337	SRS17129857	Panama
P.S.6.20.22.6	PSU-5383	PRJNA357723	SAMN33902338	SRS17129858	Panama
P.S.6.20.22.7	PSU-5384	PRJNA357723	SAMN33902339	SRS17129859	Baildon
P.S.6.20.22.8	PSU-5408	PRJNA357723	SAMN37513831	SRR26146457	Panama
P.S.6.22.22.1	PSU-5385	PRJNA357723	SAMN33902340	SRS17129860	Panama
P.S.6.22.22.2	PSU-5386	PRJNA357723	SAMN33902341	SRS17129861	Panama
P.S.6.22.22.3	PSU-5387	PRJNA357723	SAMN33902342	SRS17129862	Senftenberg
P.S.6.22.22.4	PSU-5388	PRJNA357723	SAMN33902343	SRS17129863	Panama
P.S.6.22.22.5	PSU-5389	PRJNA357723	SAMN33902344	SRS17129865	Panama
P.S.6.22.22.6	PSU-5390	PRJNA357723	SAMN33902345	SRS17129866	Panama
P.S.6.22.22.7	PSU-5391	PRJNA357723	SAMN33902346	SRS17129867	Panama
P.S.6.22.22.8	PSU-5392	PRJNA357723	SAMN33902347	SRS17129868	Panama
P.S.6.27.22.1	PSU-5393	PRJNA357723	SAMN33902348	SRS17129869	Oranienburg
P.S.6.27.22.2	PSU-5409	PRJNA357723	SAMN37513832	SRR26146456	Oranienburg
P.S.6.29.22.1	PSU-5395	PRJNA357723	SAMN33902350	SRS17129871	Montevideo
P.S.6.29.22.2	PSU-5394	PRJNA357723	SAMN33902349	SRS17129870	Oranienburg
P.S.6.29.22.4	PSU-5410	PRJNA357723	SAMN37513833	SRR26146455	Montevideo
C.S.6.29.22.1	PSU-5395	PRJNA357723	SAMN33902350	SRR23956632	Montevideo
