# Supplementary material for: Outbreak-associated Salmonella Baildon found in wastewater demonstrates how sewage monitoring can supplement traditional disease surveillance
Source: J Clin Microbiol. 2024 Sep 19;62(10):e00825-24. doi: 10.1128/jcm.00825-24 (PMC11481576; doi:10.1128/jcm.00825-24)
Supplement: Table S2 — Outbreak-associated Salmonella Baildon detected in wastewater and clinical isolates. [file jcm.00825-24-s0002.pdf]

Supplemental Table 2: Outbreak-Associated *Salmonella* Baildon Detected in Wastewater and Clinical Isolates

#Strain	Isolate identifiers	Run	Collection	Serov	Create date	SNP cluster	Locati	Isolate	Isolation source	Min-sa	Min-diff
**PNUSAS273487**	PNUSAS273487,"SRS13188836"	SRR19417524			2022-05-26T18:33:1	PDS000001722.429	USA	PDT001315600.1	human	0	0
**PNUSAS273490**	PNUSAS273490,"SRS13188845"	SRR19417533			2022-05-26T18:33:1	PDS000001722.429	USA	PDT001315604.1	human	0	0
**PNUSAS273489**	PNUSAS273489,"SRS13188914"	SRR19417602			2022-05-26T18:33:2	PDS000001722.429	USA	PDT001315611.1	human	1	1
**PNUSAS273491**	PNUSAS273491,"SRS13188944"	SRR19417632			2022-05-26T18:33:2	PDS000001722.429	USA	PDT001315619.1	human	1	1
**PNUSAS273488**	PNUSAS273488,"SRS13189374"	SRR19418063			2022-05-26T19:36:0	PDS000001722.429	USA	PDT001315638.1	human	0	0
PNUSAS273537	PNUSAS273537,"SRS13190931"	SRR19419658			2022-05-26T20:32:0	PDS000001722.429	USA	PDT001315734.1	human	1	1
**PNUSAS273534**	PNUSAS273534,"SRS13190933"	SRR19419660			2022-05-26T20:32:0	PDS000001722.429	USA	PDT001315736.1	human	1	1
PNUSAS273830	PNUSAS273830,"SRS13272421"	SRR19512122			2022-06-02T00:39:	PDS000001722.429	USA	PDT001321331.1	human	0	0
**PNUSAS273854**	PNUSAS273854,"SRS13272422"	SRR19512123			2022-06-02T00:39:	PDS000001722.429	USA	PDT001321332.1	human	0	0
**PNUSAS273848**	PNUSAS273848,"SRS13272427"	SRR19512128			2022-06-02T00:39:	PDS000001722.429	USA	PDT001321337.1	human	0	0
**PNUSAS273840**	PNUSAS273840,"SRS13272428"	SRR19512129			2022-06-02T00:39:	PDS000001722.429	USA	PDT001321338.1	human	0	0
**PNUSAS273839**	PNUSAS273839,"SRS13272429"	SRR19512130			2022-06-02T00:39:	PDS000001722.429	USA	PDT001321339.1	human	0	0
**PNUSAS273847**	PNUSAS273847,"SRS13272434"	SRR19512135			2022-06-02T00:39:	PDS000001722.429	USA	PDT001321344.1	human	0	0
**PNUSAS273851**	PNUSAS273851,"SRS13272436"	SRR19512137			2022-06-02T00:39:	PDS000001722.429	USA	PDT001321346.1	human	0	0
**PNUSAS273841**	PNUSAS273841,"SRS13272439"	SRR19512140			2022-06-02T00:39:	PDS000001722.429	USA	PDT001321349.1	human	0	0
**PNUSAS273835**	PNUSAS273835,"SRS13272459"	SRR19512160			2022-06-02T00:40:	PDS000001722.429	USA	PDT001321369.1	human	2	2
**PNUSAS273829**	PNUSAS273829,"SRS13272463"	SRR19512164			2022-06-02T00:40:	PDS000001722.429	USA	PDT001321373.1	human	0	0
**PNUSAS273832**	PNUSAS273832,"SRS13272510"	SRR19512211			2022-06-02T00:40:1	PDS000001722.429	USA	PDT001321388.1	human	0	0
**PNUSAS273852**	PNUSAS273852,"SRS13272513"	SRR19512214			2022-06-02T00:40:1	PDS000001722.429	USA	PDT001321391.1	human	0	0
**PNUSAS273849**	PNUSAS273849,"SRS13272935"	SRR19512636			2022-06-02T00:40:1	PDS000001722.429	USA	PDT001321401.1	human	1	1
**PNUSAS273844**	PNUSAS273844,"SRS13272938"	SRR19512639			2022-06-02T00:40:1	PDS000001722.429	USA	PDT001321404.1	human	0	0
**PNUSAS273853**	PNUSAS273853,"SRS13272942"	SRR19512643			2022-06-02T00:40:1	PDS000001722.429	USA	PDT001321408.1	human	0	0
**PNUSAS273836**	PNUSAS273836,"SRS13272949"	SRR19512650			2022-06-02T00:40:2	PDS000001722.429	USA	PDT001321415.1	human	0	0
**PNUSAS273845**	PNUSAS273845,"SRS13272966"	SRR19512667			2022-06-02T00:40:2	PDS000001722.429	USA	PDT001321432.1	human	0	0
**PNUSAS274674**	PNUSAS274674,"SRS13291546"	SRR19537174			2022-06-07T17:44:3	PDS000001722.429	USA	PDT001322461.1	human	0	0
**PNUSAS274673**	PNUSAS274673,"SRS13291548"	SRR19537176			2022-06-07T17:44:4	PDS000001722.429	USA	PDT001322463.1	human	0	0
**PNUSAS274672**	PNUSAS274672,"SRS13291550"	SRR19537178			2022-06-07T17:44:4	PDS000001722.429	USA	PDT001322465.1	human	0	0
**PNUSAS274680**	PNUSAS274680,"SRS13291556"	SRR19537184			2022-06-07T17:44:4	PDS000001722.429	USA	PDT001322471.1	human	0	0
**PNUSAS274675**	PNUSAS274675,"SRS13291561"	SRR19537189			2022-06-07T17:44:4	PDS000001722.429	USA	PDT001322476.1	human	0	0
**PNUSAS274898**	PNUSAS274898,"SRS13298600"	SRR19545401			2022-06-07T17:46:2	PDS000001722.429	USA	PDT001322811.1	human	0	0
**PNUSAS274892**	PNUSAS274892,"SRS13298612"	SRR19545413			2022-06-07T17:46:3	PDS000001722.429	USA	PDT001322815.1	human	1	1
**PNUSAS274890**	PNUSAS274890,"SRS13299204"	SRR19546030			2022-06-07T17:46:4	PDS000001722.429	USA	PDT001322846.1	human	0	0
**PNUSAS274899**	PNUSAS274899,"SRS13299430"	SRR19546256			2022-06-07T17:46:4	PDS000001722.429	USA	PDT001322863.1	human	0	0
**PNUSAS275803**	PNUSAS275803,"SRS13344890"	SRR19594429			2022-06-09T15:48:3	PDS000001722.429	USA	PDT001326124.1	human	1	1
**PNUSAS276036**	PNUSAS276036,"SRS13361970"	SRR19612803			2022-06-10T14:35:1	PDS000001722.429	USA	PDT001326635.1	human	0	0
**PNUSAS277273**	PNUSAS277273,"SRS13413568"	SRR19670710			2022-06-16T15:51:5	PDS000001722.429	USA	PDT001329703.1	human	0	0
**PNUSAS277276**	PNUSAS277276,"SRS13413590"	SRR19670733			2022-06-16T15:51:5	PDS000001722.429	USA	PDT001329706.1	human	1	1
**PNUSAS279524**	PNUSAS279524,"SRS13613946"	SRR19882248			2022-06-28T18:43:	PDS000001722.429	USA	PDT001348774.1	human	0	0
**PNUSAS279509**	PNUSAS279509,"SRS13613948"	SRR19882249			2022-06-28T18:43:	PDS000001722.429	USA	PDT001348775.1	human	0	0
**PNUSAS283304**	PNUSAS283304,"SRS13894078"	SRR20220042			2022-07-15T17:38:3	PDS000001722.429	USA	PDT001360690.1	human	0	0
**PNUSAS283307**	PNUSAS283307,"SRS13894082"	SRR20220046			2022-07-15T17:38:3	PDS000001722.429	USA	PDT001360694.1	human	0	0
**PNUSAS294675**	PNUSAS294675,"SRS14806462"	SRR21230238			2022-08-27T01:44:3	PDS000001722.429	USA	PDT001400187.1	human	1	1
**PNUSAS315012**	PNUSAS315012,"SRS15690537"	SRR22214388			2022-11-07T16:57:31	PDS000001722.429	USA	PDT001479878.1	human	1	1
**PNUSAS324134**	PNUSAS324134,"SRS16284494"	SRR22895702			2022-12-27T18:39:4	PDS000001722.429	USA	PDT001549093.1	human	0	0
**PNUSAS329961**	PNUSAS329961,"SRS16621630"	SRR23270643			2023-01-30T19:04:0	PDS000001722.429	USA	PDT001596886.1	human	3	3
**PSU-5372**	C.S.6.15.22.2,"PSU-5372","SRS17129872"	SRR2395663	2022		2023-03-24T19:48:	PDS000001722.429	USA:PA	PDT001667782.1	wastewater	0	0
**PSU-5384**	P.S.6.20.22.7,"PSU-5384","SRS17129859"	SRR2395664	2022		2023-03-24T19:48:	PDS000001722.429	USA:PA	PDT001667795.1	wastewater	0	0
**PSU-5370**	C.S.6.16.22.4,"PSU-5370","SRS17129853"	SRR2395665	2022		2023-03-24T19:48:	PDS000001722.429	USA:PA	PDT001667801.1	wastewater	0	0
**PSU-5369**	C.S.6.16.22.3,"PSU-5369","SRS17129852"	SRR2395665	2022		2023-03-24T19:48:	PDS000001722.429	USA:PA	PDT001667802.1	wastewater	0	0
**PSU-5401**	P.S.6.13.22.4,"PSU-5401","SRS17289373"	SRR2414228	2022		2023-04-12T16:47:3	PDS000001722.429	USA:PA	PDT001687798.1	wastewater	0	0
**PSU-5400**	P.S.6.13.22.3,"PSU-5400","SRS17289372"	SRR2414228	2022		2023-04-12T16:47:3	PDS000001722.429	USA:PA	PDT001687799.1	wastewater	0	0
**PSU-5399**	P.S.6.15.22.6,"PSU-5399","SRS17289371"	SRR2414228	2022		2023-04-12T16:47:3	PDS000001722.429	USA:PA	PDT001687800.1	wastewater	0	0
**PNUSAS348318**	PNUSAS348318,"SRS17629679"	SRR24520323			2023-05-12T15:51:53	PDS000001722.429	USA	PDT001729585.1	human	0	0
**PSU-5402**	C.S.6.16.22.1,"PSU-5402","SRS18954242","	SRR2614646	2022-06-16		2023-09-22T20:00:	PDS000001722.429	USA:PA	PDT001893749.1	wastewater	0	0
